# Availability, Affordability, and Accessibility of a Healthful Diet in a Low-Income Community, Central Falls, Rhode Island, 2007-2008

**Published:** 2010-02-15

**Authors:** Kim M. Gans, Marissa Sheldon, Ryan Tai, Tiffiney George, Deborah N. Pearlman, Eliza Lawson

**Affiliations:** Brown University Institute for Community Health Promotion; University of North Carolina, School of Public Health, Chapel Hill, North Carolina. At the time of this study, Ms Sheldon was affiliated with the Department of Community Health, Brown University, Providence, Rhode Island; Brown University Medical School, Providence, Rhode Island; Brown University Public Health Program, Providence, Rhode Island; Brown University Public Health Program, Providence, Rhode Island; Rhode Island Department of Health, Providence, Rhode Island

## Abstract

**Background:**

Many Americans have diets that do not meet the dietary guidelines set by the US Department of Agriculture (USDA). Additionally, low-income people have the highest rates of obesity and have difficulty accessing the necessary foods for maintaining a healthful diet.

**Context:**

In December 2007 and January 2008, 21 retail food stores in Central Falls, Rhode Island, where residents were predominantly low-income Hispanics, were evaluated for the availability and costs of foods that fulfill the USDA's Thrifty Food Plan (TFP) guidelines.

**Methods:**

Each surveyed store was evaluated for variety and weekly cost of 3 different types of market baskets (2 families and an elder). Each store's proximity to public transportation was estimated by using geographic information systems mapping.

**Outcome:**

Only 2 stores in Central Falls and the discount supermarket in an adjacent city, Pawtucket, carried enough variety of foods to fill the TFP basket. At the 2 stores, costs were up to 40% higher, and at the discount store, costs were up to 18% cheaper, than the national average. Each of the stores was accessible by public transportation.

**Interpretation:**

Meeting the USDA TFP guidelines is difficult in this low-income, predominantly Hispanic city. Although the components of the TFP are available, high prices may make a nutritious diet unaffordable.

## Background

Many Americans have diets that do not meet the national dietary guidelines (1,2), which threatens public health because failure to meet dietary recommendations is associated with an increased risk of obesity and related chronic diseases and risk factors (1-3). In the United States, Hispanic men and women are more likely to be obese than their non-Hispanic white counterparts (4). Additionally, populations with the highest rates of poverty also have the highest rates of obesity (1,5).

Accessing the necessary foods for maintaining a healthful diet can be especially challenging for low-income people. Social, economic, and personal factors, such as lack of knowledge or interest in healthy eating, also impede access to healthful foods. To save money, people living in poverty are likely to limit or remove more expensive food items, such as fresh produce from their diets and to consume energy-dense foods with a high fat and sugar content (5,6).

Dietary choices may also be influenced by the availability of retail food stores and fast-food restaurants in a person's area of residence (7). Food deserts are geographic regions where few or no grocery stores exist (8). Inner-city, low-income, and primarily African American neighborhoods are more likely to be characterized as food deserts (9,10). Overall, the presence of accessible grocery stores is directly related to the prevalence of overweight, obesity, and hypertension (8).

## Context

In December 2007 and January 2008, we analyzed the availability and affordability of a healthy market basket (Appendix) in Central Falls, a Rhode Island city in which 40.8% of children live in poverty (11). Given the demographics of Central Falls, living in this city may increase the risk of obesity or food insecurity (limited or uncertain availability of or access to food). According to 2000 US census data, 22.8% of households in Central Falls had an annual income of less than $10,000, and the median household income was $22,628 (12). Central Falls is also composed of a largely Hispanic community, with 47.8% of residents in 2000 identifying as Hispanic or Latino (12) (Table). Overweight and obesity data from the Special Supplemental Nutrition Program for Women, Infants, and Children (WIC) show that WIC children (aged 2-5) in Central Falls have the highest rates of obesity in Rhode Island (13). In Rhode Island, Hispanic adults and children have higher rates of obesity than non-Hispanic whites or blacks (among children, 33% of Hispanics vs 14% of non-Hispanic whites or blacks; among adults, 62% of Hispanics vs 56% of non-Hispanic whites or blacks). Rhode Island residents with lower incomes are also more prone to obesity; 20% of adults aged 18 or older with incomes less than $15,000 are obese, compared with 15% of adults with incomes greater than $50,000 (unpublished data, Eliza Lawson, Rhode Island Department of Health, April 16, 2008).

## Methods

Two methods can be used to evaluate the food landscape of a community: 1) performing an environmental scan, an audit of the community environment that evaluates what food resources are available; or 2) performing a market basket analysis. The term "market basket" refers to a grocery list of foods that will fulfill the US Department of Agriculture (USDA) dietary recommendations. A market basket analysis evaluates whether or not each food item on the list is sold at a particular store, the price of each available item, and the nutritional quality of the foods (eg, low-fat/low-calorie vs original version, whole-grain vs white bread, rice, pasta). Studies that use an environmental scan (9) can help identify food deserts and other disparities that contribute to the eating habits of people in particular regions. Studies that use a market basket as the basis of analysis (10,14) focus more specifically on the cost associated with eating well in a particular community. We combined aspects of both methods to more precisely assess the overall accessibility, availability, and affordability of a healthy diet in Central Falls.

This study used the USDA Thrifty Food Plan (TFP) as the framework to determine the adequate quantity and quality of foods purchased at minimal cost to meet the nutritional guidelines outlined in the food guide pyramid (15). The TFP provides recommendations for a weekly market basket to feed several different age and sex categories. We determined the quantities of each food needed to feed 2 distinct 4-person family models based on the TFP recommendations. One family comprised a mother aged 19-50, a father aged 19-50, a child aged 2-3, and a child aged 4-5. The other family consisted of a mother aged 19-50, a father aged 19-50, and 2 school-aged children, aged 9-11 and aged 12 or 13. The TFP does not differentiate between boys and girls for children up to age 11. For the 12- or 13-year-old, however, the food quantities for boys and girls differ. For the ease of our study analyses, we averaged the given amounts for a boy and a girl and used these quantities for the 12- or 13-year-old child. We also evaluated the price of the TFP for a single, elderly (aged >71 years) woman.

The Rhode Island Department of Health's Initiative for a Healthy Weight (IHW) collected data at 21 retail food stores in Central Falls during the summer of 2007; these original data helped us decide which foods to include on our list. We constructed a market basket of 58 food items that were obtainable in Central Falls and complied with the TFP guidelines (Appendix). Foods that IHW found to be generally unavailable in Central Falls, such as frozen entrees, bagels, pastries, wild rice, and frozen chicken, were excluded from our adapted TFP market basket. Fresh fruits and vegetables must be available year-round to be included in the market basket. We excluded food items such as condiments, spices, sweets, coffee, and tea because these products are generally used in small quantities on a weekly basis.

For this study, the same 21 retail food stores were surveyed during December 2007 and January 2008. The sample included 9 small grocery stores, 8 convenience stores, 3 bakeries, and 1 meat market. Because no large supermarkets exist in Central Falls, we evaluated a discount supermarket that was on the Central Falls-Pawtucket town line. We surveyed each store to determine the availability of each food item in our adapted TFP market basket. The item was considered available if the store had it on the shelves in any brand or package size. For each item that was present in the store, we recorded the unit price of the cheapest brand and package size. If a unit price was not given, the price and package size were both noted and later converted to a price (per pound).

The cost of each family-adjusted TFP market basket was measured under 2 conditions: 1) a cumulative average cost across all 21 stores, and 2) a cost for each individual store in which a complete market basket could be purchased. We compared these prices to the national average cost of the TFP for December 2007 (16). To calculate the total cost of the market basket, all prices were converted to prices per pound. If fruits or vegetables were priced per piece, rather than by the pound, we weighed the produce to determine a weight conversion. Liquid measurements were also converted from fluid ounces to pounds. When multiple forms of the same food were evaluated (eg, 1% milk, skim milk), the average price was calculated to use in the total market basket cost. For the aggregate cost in Central Falls, the prices of each food item were averaged across all stores in which they were available. We calculated the prices of the market baskets for the 2 defined families by multiplying the price per pound of each food by the quantity (in pounds per week) specified in the TFP on the basis of the age and sex of each family member.

We also evaluated the accessibility of the food stores relative to the Rhode Island Public Transit Authority (RIPTA) bus routes and stops by using geographic information systems (GIS) mapping, a process that assesses spatial relationships (17). The stores were integrated within ArcGIS (ESRI, Redlands, California) by geocoding addresses and comparing these with the locations of public transportation routes and stops. Stores were considered accessible to residents if they were within 1 block of a bus or trolley stop.

## Outcome

### Food availability

Many staple foods were commonly found throughout Central Falls. Among the most frequently sold items were white bread, white rice, white pasta, any variety of tomatoes, milk, juice, eggs, and any variety of fish. The less readily available foods were often healthier items such as brown rice, broccoli, green leaf lettuce, and fresh meats. Of the 21 stores, 1 small grocery store (store A) and 1 convenience store (store B) were the only vendors in Central Falls that sold a full market basket that followed the TFP guidelines. The discount supermarket in Pawtucket (store C) also sold all of the foods on our list. The GIS map demonstrated that Central Falls residents without a car could still access most of the stores through public transportation.

### Food costs

Compared with the national average cost per week, the aggregate cost of the market basket across all Central Falls retailers was approximately 41% higher; the prices of TFP baskets at stores A and B were 30% and 41% higher than the national average, respectively (Figure). Again, the costs of our market baskets do not include infrequently used items such as condiments, but the national average cost includes these items. Therefore, the true market basket costs in Central Falls are higher than reported. At store C, the market basket costs were 9% to 18% lower than the national average, and as much as 42% lower than the Central Falls average (Figure).

**Figure.**
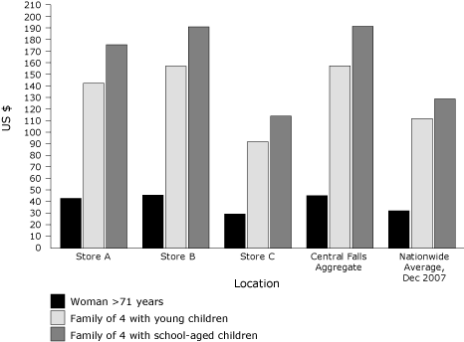
Comparison of Thrifty Food Plan (TFP) market basket costs by store type and family composition, Rhode Island, 2007-2008. Stores A and B are grocery stores in Central Falls. Store C is a discount supermarket in neighboring Pawtucket.

**Table:** 

**Location**	**Family Composition/Market Basket Cost (US$)**		

	**Woman (aged >71)**	**Family of 4 with Young Children**	**Family of 4 with School-aged Children**
Store A	43.34	143.19	176.65
Store B	46.18	157.99	192.03
Store C	29.81	92.59	114.62
Central Falls aggregate	45.67	158.19	192.58
Nationwide average, December 2007	32.60	112.60	129.60

### Food affordability

The data indicate that the average resident of Central Falls would only be able to afford all necessary food items in the TFP by shopping at store C in Pawtucket. If residents are unable to walk or use public transportation to travel to store C, all foods necessary for a healthy diet on the basis of the TFP could also be purchased at stores A and B, but at a much higher cost. The TFP is used to determine Food Stamp Program benefits (14). Consequentially, the TFP should be affordable to those who receive assistance from the Food Stamp Program, but this is not the case in Central Falls. The monetary value of food stamps is equal to the maximum value per household ($542 per month for a 4-person household, $162 for a single-person household), less 30% of the net household income (18). According to the data gathered in Central Falls, a family of 4 with 2 young children pays $158.46 per week for the TFP, which translates to $679.11 per month; a family of 4 with 2 older children pays $825.34 per month; and a single elderly woman pays $195.73 per month. These prices do not take into account the costs of infrequently used items such as condiments. Therefore, even with the maximum food stamp allotment, which is only awarded to households with no net income, a family of 4 may have to spend as much as $283 (or more) of its own resources on food each month. These results are similar to findings of a Boston Medical Center study showing that the monthly cost of a healthy diet on the basis of TFP in Boston, Massachusetts, is $148 more than the maximum food stamp benefit (19).

## Interpretation

### Impact

The results of our research support the findings of previous studies that also examined the nutritional environments of low-income communities (9,10,14). Central Falls residents have limited access to TFP items; when healthy foods are available within the city, residents of Central Falls have to pay more than the average American for a healthy diet.

The primary barrier to purchasing an affordable healthy market basket in Central Falls is the lack of a supermarket in the community. Previous studies found that convenience stores and small grocery stores outnumber supermarkets in poorer areas; smaller, nonchain stores have limited varieties of foods at higher prices, especially for healthier foods (6,9,10). Store C in Pawtucket, in comparison, is a discount chain supermarket; accordingly, it had substantially lower prices than any of the stores in Central Falls.

Foods that are readily available and affordable to the Central Falls community often do not include healthy items recommended in the TFP, such as fresh fruits and vegetables. Consequentially, many Central Falls residents may not purchase and consume foods of a high nutritional quality. Because a poor-quality diet is linked to obesity and related chronic diseases (4), people in Central Falls may be at higher risk of developing chronic health problems.

To purchase a healthful diet at an affordable price, the easiest solution for Central Falls residents is to travel to store C in the neighboring city of Pawtucket. Store C lies directly on a RIPTA bus line; thus, those who have a bus pass should be able to commute to the supermarket. The bus line that passes store C only runs through a small segment of Central Falls; residents who do not live directly on this route must walk to the nearest bus stop or switch buses to travel from their home to store C. These potential barriers could limit the amount of groceries that people in Central Falls would be able to carry in 1 trip.

Data from this case study suggest that low-income residents of Central Falls live in a nutritionally inadequate environment. As a result, they may have to either pay more for healthy food or suffer the health consequences of eating a poorer quality diet. Prioritizing financial needs over nutrition may contribute to the high rates of obesity among Central Falls residents. Efforts are under way to publicize the study's findings, and as a result of the study, interventions are being developed, including establishment of a discount produce market in Central Falls.

### Directions for future research

Because of time constraints, numerous foods included in the TFP were not assessed. For example, within the grains category, we did not look at crackers, chips, pastries, or bagels, but instead focused on breads, cereals, and pastas. Nonetheless, we are confident the items that were assessed are representative of a healthy diet and adequately reflect the general prices of food in the city. Additionally, the data gathered only reflect the foods that are both included in the TFP and available at retail food stores in Central Falls. The TFP was used as a model for a healthy diet, but there are no survey data available indicating residents' personal preferences. Given more resources and time, we would survey residents to determine actual food consumption and include a more comprehensive list of food items to survey.

The methods and framework of this case study can be applied to other low-income communities to determine access and affordability of the TFP. Assessing food access and affordability for high-risk communities is a first step to initiating health promotion interventions.

## Figures and Tables

**Table.** Demographic Characteristics of Residents, Central Falls, Rhode Island, 2000 US Census

**Characteristic**	No.	Percent
**Total population**	18,928	100.0
**Ethnicity**		
Hispanic or Latino (of any race)	9,041	47.8
Not Hispanic or Latino	9,887	52.2
**Household composition**		
Family households with own children <18 years	2,607	38.9
Married-couple families with own children <18 years	1,340	20.0
Householder living alone, ≥65 years	848	12.7
Average family size	3.38	NA
**Household income in 1999**		
Households with <$10,000 household income	1,529	22.8
Median household income ($)	22,628	NA
**Poverty status^a^ in 1999**		
Families living below poverty level	1,147	25.9
Families with related children aged <18 years living below poverty level	988	34.6
Families with related children aged <5 years living below poverty level	547	40.9

Abbreviation: NA, not applicable.

a Poverty status is defined by a total family income less than a predetermined poverty threshold, which is a dollar amount specific to the size and age composition of the family. For people who do not live with family members, the poverty threshold is based on individual income (http://www.census.gov/hhes/www/poverty/povdef.html).

## Appendix. Market Basket for Case Study, Central Falls, Rhode Island, 2007-2008

### Grains

- Whole grain bread
- Brown rice
- Oatmeal
- Whole grain cereal
- Popcorn
- White bread
- White rice
- White pasta
- Non-whole grain cereal

### Vegetables

- Whole potatoes
- Instant mashed potatoes
- Frozen french fries
- Green bell peppers
- Broccoli (fresh or frozen)
- Green leaf lettuce
- Carrots (fresh, frozen, or canned)
- Black beans (canned or dried)
- Lentils (canned or dried)
- Canned baked beans
- Tomatoes (fresh or canned whole, diced, or sauce)
- Corn (frozen or canned)
- Cabbage

### Fruits

- Bananas
- Apples
- Oranges (fresh or canned Mandarin)
- Pears (fresh or canned)
- 100% juice

### Milk products

- Whole milk
- 1% or skim milk
- Yogurt
- American cheese

### Meats and beans

- Ground beef — lean and standard
- Fresh whole chicken
- Chicken breasts
- Frozen fish
- Canned tuna
- Hot dogs
- Deli turkey breast
- Peanut butter
- Eggs

### Other foods

- Vegetable oil
- Coke
- Diet Coke
- Fruit drinks
- Canned condensed soup
